# Sorafenib with interleukin-2 *vs* sorafenib alone in metastatic renal cell carcinoma: the ROSORC trial

**DOI:** 10.1038/bjc.2011.103

**Published:** 2011-03-29

**Authors:** G Procopio, E Verzoni, S Bracarda, S Ricci, C Sacco, L Ridolfi, C Porta, R Miceli, N Zilembo, E Bajetta

**Affiliations:** 1 Department of Medical Oncology, Unit 2, Fondazione IRCCS Istituto Nazionale dei Tumori, Via G. Venezian, Milan 1-20133, Italy; 2 Ospedale San Donato, USL 8, Arezzo, Italy; 3 O.M. Santa Chiara, Pisa, Italy; 4 A.O. Udine, Udine, Italy; 5 Istituto Scientifico Romagnolo per lo Studio e la Cura dei Tumori (I:R:S:T), Meldola, Italy; 6 IRCCS San Matteo University Hospital Foundation, Pavia, Italy; 7 Unit of Clinical Epidemiology and Trial Organization, Fondazione IRCCS Istituto Nazionale dei Tumori of Milan, Milan, Italy; 8 Policlinico di Monza, Monza, Italy

**Keywords:** sorafenib, interleukin-2, targeted therapies, renal cell carcinoma, immunotherapy

## Abstract

**Background:**

Preclinical investigations support combining sorafenib with IL-2 in the treatment of metastatic renal cell carcinoma (mRCC).

**Methods:**

In this open-label, phase II study, 128 patients with mRCC were randomised to receive oral sorafenib, 400 mg twice daily, plus subcutaneous IL-2, 4.5 million international units (MIU) five times per week for 6 in every 8 weeks, or sorafenib alone. After enrolment of the first 40 patients, IL-2 dose was reduced to improve the tolerability.

**Results:**

After a median follow-up of 27 months, median progression-free survival (PFS) was 33 weeks with sorafenib plus IL-2, and 30 weeks with sorafenib alone (*P*=0.109). For patients receiving the initial higher dose of IL-2, median PFS was 43 weeks *vs* 31 weeks for those receiving the lower dose. The most common adverse events were asthenia, hand–foot syndrome, hypertension, and diarrhoea. Grade 3–4 adverse events were reported for 38 and 25% of patients receiving combination and single-agent treatment, respectively.

**Conclusion:**

The combination of sorafenib and IL-2 did not demonstrate improved efficacy *vs* sorafenib alone. Improvements in PFS appeared greater in patients receiving higher-dose IL-2.

For the past two decades, immunotherapy has been the only therapeutic approach to demonstrate a moderate, but unequivocal benefit in a restricted patient population with clear-cell renal cell carcinoma (Yang *et al*, 2003; McDermott *et al*, 2005). Indeed, after the cloning of the genes encoding interleukin (IL)-2 and interferon-alpha (IFN-*α*), extensive clinical investigations undertaken in metastatic renal cell carcinoma (mRCC) showed that these biological agents yielded response rates ranging from 8 to 26% with only modest effects on the natural history of the disease in terms of overall survival (OS) (McDermott and Atkins, 2008). In a phase II non-randomised trial, Fisher *et al* (2000) reported long-term complete remissions with high doses of IL-2 administered intravenously, thus suggesting that the efficacy of IL-2 might correlate with doses and administration methods.

The multikinase inhibitor (TKI) sorafenib (Nexavar; Bayer HealthCare, Milan, Italy), which targets the Raf/MEK/ERK pathway as well as vascular endothelial growth factor receptors (VEGFRs) 1, 2 and 3, platelet-derived growth factor receptor (PDGFR)-b, c-Kit, Fit-3 and RET, has shown dual anti-proliferative and anti-angiogenic activity (Wilhelm *et al*, 2004). A pivotal, randomised, placebo-controlled, phase III clinical trial (the TARGET study) (Escudier *et al*, 2007a) demonstrated a significant improvement in progression-free survival (PFS) with sorafenib (5.5 months) *vs* placebo (2.8 months; *P*<0.001) in patients with clear-cell mRCC refractory or intolerant to cytokines. Following this trial, the drug was the first targeted therapy to be approved by the US Food and Drug Administration for the treatment of this disease. A subsequent randomised phase two trial comparing sorafenib with IFN-*α* as first-line treatment in mRCC demonstrated no differences in terms of PFS in the two arms of therapy (5.7 *vs* 5.6 months, *P*=0.50) (Escudier *et al*, 2009).

Preclinical investigations have provided evidence to support the combination of sorafenib with IL-2 (Iguchi *et al*, 2009; Amagai *et al*, 2010). IL-2 acts to induce the proliferation and activation of T cells, B cells, natural killer (NK)-cells and lymphokine-activated killer cells, resulting in multiple biological effects including the proliferation of antigen-stimulated T cells and the induction of cytotoxicity through the activation of tumouricidal monocytes. This led to the hypothesis that the different mechanisms of action of sorafenib and IL-2 on T-cell signalling and proliferation could be synergistic, and provide improved clinical outcomes in patients with mRCC.

On the basis of these assumptions, this randomised, prospective, phase II, clinical study compared the combination of sorafenib plus IL-2 *vs* sorafenib alone in patients with mRCC not previously treated with systemic therapy.

## Patients and methods

### Patients

Eligible patients were aged 18 years or older, with a life expectancy of at least 3 months, and an Eastern Cooperative Oncology Group (ECOG) performance status of two or less. They were required to have a histologically confirmed diagnosis of advanced or metastatic RCC, all histologies, with at least one measurable unidimensional lesion detected by computed tomography (CT) or magnetic resonance imaging (MRI) scan and evaluated according to Response Evaluation Criteria for Solid Tumours (RECIST) criteria version 1.0 (Therasse *et al*, 2000). In cases of initial diagnosis of RCC dating back more than 2 years, cytohistological confirmation of RCC origin of the current lesions was mandatory. Eligible patients had not been previously treated with systemic therapy for metastatic disease, but patients could have undergone previous nephrectomy. The following baseline haematochemical values were considered mandatory for eligibility: absolute neutrophil count ⩾1.5 × 10^9^ l^−1^; platelet count ⩾100 × 10^9^ l^−1^; haemoglobin >9 g dl^−1^; serum creatinine ⩽2.0 × the upper limit of normal (ULN); total bilirubin <1.5 × ULN; aspartate aminotransferase (AST) or alanine aminotransferase (ALT) <2.5 × ULN for patients without liver metastases and <5 × ULN for patients with liver metastases; amylase and lipase <1.5 × ULN. Exclusion criteria included a history of brain metastases, presence of concomitant illnesses, or medical conditions, such as serious respiratory or cardiovascular diseases, unstable angina, uncontrolled hypertension (⩾160 mm Hg systolic and/or 90 mm Hg diastolic pressure), unstable diabetes mellitus, serious bacterial or fungal infections, or potentially life-threatening autoimmune disorders. Patients with other previous malignancies were considered ineligible, with the exception of those with a history of adequately treated basal- or squamous-cell skin cancer or *in situ* cervical cancer.

### Study design

This was a prospective, randomised, open-label, multicentre, phase II study designed to evaluate the efficacy and safety of the combination of sorafenib plus IL-2 *vs* sorafenib alone in previously untreated patients with unresectable or metastatic RCC. The primary endpoint of the study was PFS, and the secondary endpoints included objective response rate (ORR), OS, and the safety profile of the two therapeutic regimens.

Patients were randomly allocated (1 : 1) to treatment with either oral sorafenib 400 mg (2 × 200 mg tablets) twice daily for the entire study period combined with IL-2 administered subcutaneously at a dose of 4.5 million international units (MIU) on 5 days per week for 6 weeks with treatment repeated every 8 weeks, or with sorafenib alone at the same dose as above. However, after treatment of the first 40 patients, of whom 20 were randomised to the combination treatment arm, the protocol was amended to reduce the dose of IL-2–3 MIU 5 days per week, 2 weeks on and 2 weeks off, because of the onset of AEs. Patients received study treatment until tumour progression, symptomatic deterioration, or onset of unacceptable toxicity requiring drug discontinuation and withdrawal of the patient from the study.

The study design was approved by the Ethical Committees of each Institution and was performed in accordance with the Declaration of Helsinki and Good Clinical Practice guidelines. At enrolment, each patient gave written informed consent. Randomisation was performed centrally at the Italian Trials in Medical Oncology (ITMO) office. To ensure balance between the treatment arms with respect to centre, Memorial Sloan-Kettering Cancer Centre (MSKCC) risk group (low–intermediate–high) and histological type (clear cell *vs* non-clear cell), the minimisation method was applied using the Minim program (Evans *et al*, 2010, freely available at: http://www-users.york.ac.uk/~mb55/guide/minim.htm, last access: 14 September 2010). The program was set by the Unit of Medical Statistics, Biometry and Bioinformatics. ITMO staffs were involved in running the program and assigning eligible patients to treatment arm.

### Safety and efficacy assessments

Toxicity was assessed using the National Cancer Institute Common Toxicity Criteria (NCI-CTC) (version 3.0). In the case of severe toxicities (grades 3–4) that were deemed likely to be related to sorafenib treatment, such as haematological toxicity, hypertension, and skin reactions, sorafenib was reduced to a dose of 400 mg once daily or every other day, or was temporarily discontinued. If a further dose reduction was required, or if no recovery (grades 0–1) was evident after a 2-week discontinuation of sorafenib, treatment was discontinued. No dose reduction of IL-2 was initially defined in the protocol; in the case of AEs related to IL-2: drug administration was temporarily stopped and then restarted at the same dosage after AE resolution. After the protocol amendment, the occurrence of grade 3–4 AEs resulted in dose reduction of IL-2 to 2 MIU on 5 days per week, 2 weeks on and 2 weeks off. If after 2 weeks no recovery (grades 0–1) was observed, the patient was withdrawn from the study.

RECIST criteria version 1.0 was used for response assessments. Evaluations were carried out every 8 weeks during the first 24 weeks of treatment and then every 12 weeks thereafter. Tumour measurements were carried out by CT or MRI scan, with all initial diagnoses of complete and partial responses confirmed 4 weeks later.

### Statistical analysis

Sample size was calculated according to a phase 2.5 design (Simon *et al*, 2001) considering PFS as endpoint (progression or death without progression, whichever occurred first). Assuming exponentially distributed time and 10% significance level (one-sided log-rank test) to detect a 3-month increase in median PFS time in the experimental arm from an anticipated median of 6 months in the control arm (Escudier *et al*, 2009), 110 events yielded 80% power to detect the target difference, with a sample size of 128 patients recruited over 24 months, and with a maximum length of follow-up of 36 months.

The efficacy and safety analyses were performed on data from the intent-to-treat population. All clinical and instrumental variables and toxicity data were analysed by descriptive statistics: mean, s.d., minimum, and maximum values for continuous variables, and absolute and relative frequencies for categorical variables. Curves relevant to PFS were estimated by the Kaplan–Meier method and compared by means of the log-rank test. Reports of AEs were categorised according to type, severity, and outcome.

## Results

### Patients

**Figure 1:**
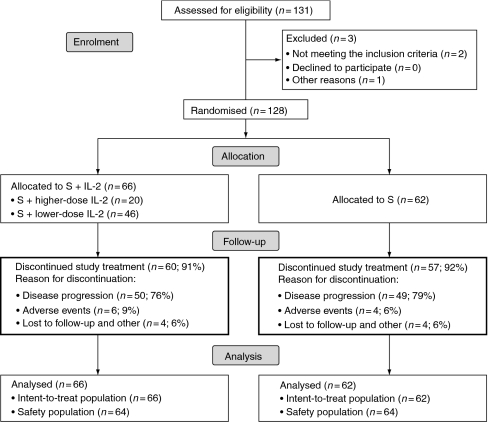
Flow of patients through the study.

From October 2006 to February 2008, 128 patients entered the study (66 receiving combination treatment with sorafenib plus IL-2, and 62 receiving sorafenib monotherapy), all of whom were included in the analyses. Four patients in both arms of treatment were unevaluable for response because of refusal or being lost to follow-up (Figure 1). Baseline characteristics of the two groups were well balanced with regards to age, sex, histology, previous surgery, tumour stage, site of metastatic disease, and risk category (Table 1). Overall 73 and 74% of all patients in the combination and single agent arms, respectively, were nephrectomised before study entry. In total, 20 (30%) and 9 (15%) patients had only lung disease in the combination and single agent arms, respectively, whereas 31 (47%) and 32 (52%) patients had multiple sites of disease in the combination and single agent arms, respectively.

Median duration of sorafenib alone or combination treatment was 29 and 35 weeks, respectively.

Medical treatment was withdrawn in 15 and 12% of patients in the combination and single agent arms, respectively, as a result of treatment refusal, AEs, or being lost to follow-up. A dose reduction was undertaken in 35 and 31% of patients in the combination and monotherapy arms, respectively.

A dose reduction in the first cohort of patients receiving sorafenib and higher dose of IL-2 was undertaken in 48% of the population because of toxicity.

### Efficacy

**Figure 2:**
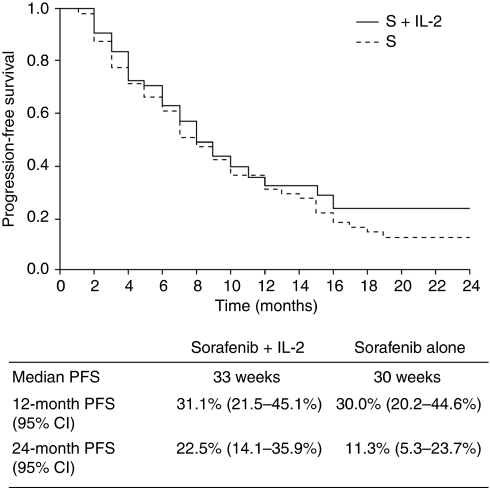
Progression-free survival curves in patients treated with the combination of sorafenib plus IL-2 (S+IL2) or sorafenib alone (S).

Median PFS was not significantly different between the two treatment groups (*P*=0.109): median PFS time was 33 weeks with the combination of sorafenib plus IL-2, compared with 30 weeks with sorafenib monotherapy. In all, 1- and 2-year PFS was 31.1% (95% CI: 21.5–45.1) and 22.5% (95% CI: 14.1–35.9), respectively, with combination therapy and 30.0% (95% CI: 20.2–44.6) and 11.3% (95% CI: 5.3–23.7) with sorafenib monotherapy (Figure 2).

**Figure 3:**
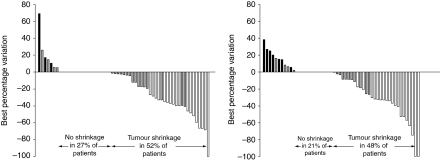
Waterfall plot showing the best variation from baseline in the sum of target lesion diameters. The investigator-assessed response is differentiated by colour (partial response, white; stable disease, grey; disease progression, black).

With combination therapy, 18 patients (27.3%) had a partial response and 35 (53.0%) had stable disease. Six patients (9.1%) had a long-term partial response during at least 12 months. With sorafenib monotherapy, 9 patients (14.5%) achieved partial response and 37 (59.7%) had stable disease. The numbers of patients with disease progression were 9 (13.6%) and 12 (19.4%) in the combination and single arms, respectively. Tumour shrinkage is illustrated in Figure 3. After a median follow-up time of 27 months, median OS was not reached in either treatment group.

**Figure 4:**
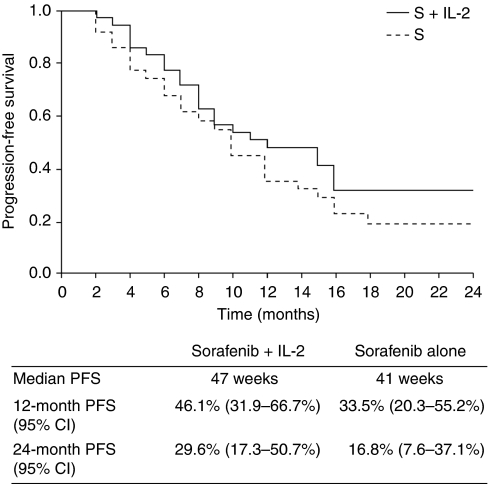
Progression-free survival curve in the low-risk subgroup of patients treated with the combination of sorafenib plus IL-2 (S+IL2) or sorafenib alone (S).

**Figure 5:**
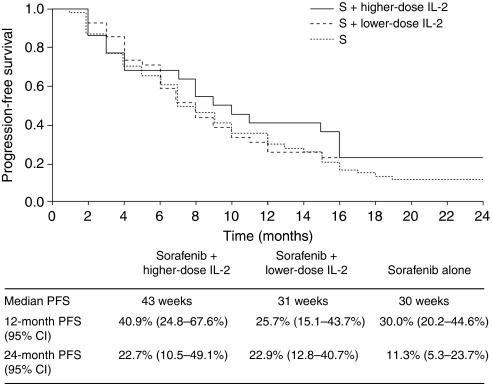
Progression-free survival curves in patients treated with the combination of sorafenib plus higher dose of IL-2 (S+higher dose IL2), sorafenib plus lower dose of IL-2 (S+lower dose IL2), or sorafenib alone (S).

The subgroup analysis demonstrated that improvement in PFS was more evident in the population with low-risk disease than in those with intermediate- or high-risk disease, with a median PFS of 47 weeks in the combination therapy group compared with 41 weeks in the sorafenib monotherapy group (Figure 4). In contrast, in the population with intermediate-risk disease, median PFS was 21 weeks in the combination therapy group compared with 29 weeks in the sorafenib monotherapy group. PFS was not calculated in the high-risk subgroup because of the low number of patients in this category. Median PFS for patients with clear-cell histotype was 36 weeks with combination therapy and 32 weeks with sorafenib monotherapy. The small number of patients with non-clear-cell histotypes did not allow efficacy evaluations in these less frequent histologies. Considering the two subgroups of patients receiving the higher (full) or lower (reduced) doses of IL-2, median PFS was 43 weeks in the higher-dose subgroup and 31 weeks in the lower-dose subgroup (Figure 5).

Considering those patients who had only lung metastases, partial responses were observed in 5 out of 20 (25%) patients in the combination therapy arm and 1 out of 9 (11%) patients in the sorafenib arm. In this limited subgroup, tumour shrinkage was overall documented in 10 out of 20 (50%) patients receiving combination treatment.

### Safety

The incidence of AEs in the sorafenib plus IL-2 combination therapy group was 80% for any grade and 38% for grade ⩾3 AEs. In the sorafenib monotherapy group, 92% of patients reported AEs of any grade and 25% reported grade ⩾3 AEs. The most common (incidence >5%) grade ⩾3 AEs (combination *vs* monotherapy) were: skin (14 *vs* 9%), gastrointestinal (8 *vs* 5%), and general disorders (8 *vs* 3%). The overall AEs are shown in Table 2. For the first 20 patients treated with higher-dose IL-2, the most common AE requiring the protocol amendment was grade >2 asthenia, which was reported in 11 patients (55%). After the amendment, the incidence and manageability of these AEs improved. Dose reduction due to AEs was undertaken in 22 patients (33%) in the combination therapy group and in 15 patients (24%) in the sorafenib monotherapy group.

## Discussion

Until recently, the prognosis of patients with mRCC was extremely poor because of the high resistance of this disease to the available therapeutic approaches, such as conventional cytotoxic chemotherapy, radiotherapy, and hormone therapy. The subsequent introduction of cytokines, particularly IL-2, produced varying efficacy and safety outcomes, raising a series of questions about the most appropriate doses and methods of administration. During recent years, advances in the understanding of the molecular biology of RCC have led to the successful development of several new anti-angiogenic factors with promising efficacy and acceptable toxicity profiles (Hudes *et al*, 2007; Escudier *et al*, 2007b; Motzer *et al*, 2007, 2008; Rini *et al*, 2010; Sternberg *et al*, 2010). This has resulted in a shift from the use of cytokine-based therapies to these newer therapeutic approaches.

The current study is, to our knowledge, the first to evaluate the combination of sorafenib and IL-2 in mRCC. When the study was planned in 2006, there was limited knowledge regarding the possible synergistic action of sorafenib plus IL-2. This trial was not based on a previous phase I study or from any extrapolation from preclinical data. Therefore, the timing and dose of drug administration, and IL-2 dose modifications were established on rather empirical assumptions, chiefly based on concerns for safety of the combination and without any specific guidance. Given that, tolerability is difficult to interpret because of the heterogeneity of IL-2 doses and schedules used.

The results of the present study failed to meet the pre-specified statistical endpoints. The trend towards a superiority of the combination treatment in terms of median PFS did not reach statistical significance. The median PFS of 33 weeks observed in patients treated with the combination compared with 30 weeks for those treated with sorafenib alone (*P*=0.109), does not appear to support a synergistic effect of sorafenib and IL-2 in the overall study population. The subgroup analysis demonstrated that the difference in PFS was more evident in the population with low-risk disease than in those with intermediate-risk disease, with a median PFS of 47 weeks *vs* 41 weeks in favour of combination therapy. These findings suggest that in some subgroups of patients with slowly progressing disease, there may be a benefit to using cytokines. In contrast, the population with intermediate-risk disease had a median PFS of 21 *vs* 29 weeks in favour of sorafenib monotherapy.

This analysis suggest a detrimental effect of the addition of IL-2 in the population with intermediate prognostic features, consistent with a previous study that showed that IL-2 treatment had no benefit in patients with intermediate-risk disease (Negrier *et al*, 2007).

After treating the first 40 patients, a reduction in IL-2 dose to 3 MIU on 5 days per week, 2 weeks on followed by 2 weeks off, was necessary because of the onset of high rates of asthenia (55% of grade 3 or 4). The resulting median PFS was 43 weeks in patients receiving the higher dose of IL-2 plus sorafenib *vs* 31 weeks in patients who received the lower dose of IL-2 plus sorafenib, suggesting that the efficacy of the combination treatment could be associated with IL-2 dose. Therefore, assuming that a synergistic effect could be recognised, factors such as route of administration, schedule, and doses of IL-2 used could be called into question as responsible for the different results. Previous investigations of IL-2 have shown that by changing the schedule of the drug by either continuous infusion or subcutaneous administration it is possible to decrease toxicity while maintaining therapeutic efficacy. Moreover, comparisons between higher and lower doses of IL-2 have shown a higher response rate with higher doses without, however, any significant survival advantage (Negrier *et al*, 1998). In contrast, in selected patients, the use of high doses of IL-2 has provided long-term complete remission (Rosenberg *et al*, 1994).

By contrast, results from the AVOREN study demonstrated that a dose reduction of IFN-*α* did not influence the activity of the combination of bevacizumab plus IFN-*α* when administered as first-line treatment in mRCC (Melichar *et al*, 2008). Previous experience suggests that the activity of IL-2 could be also related to the site of disease (Akaza *et al*, 2006). Proof that lung metastases had more benefit from low-dose IL-2 in comparison with others sites of disease (Miyake *et al*, 2009). In our trial, 5 out of 20 (25%) patients who had only lung disease developed a partial response with the combination therapy, while 1 out of 9 (12%) patients in the sorafenib arm had a partial response. The limited number of patients with these characteristics means that we cannot draw any firm conclusions concerning the usefulness of the combination of sorafenib and IL-2 in patients with only lung disease. Similar to other trials combining sorafenib±IFN-*α*, the current results support the hypothesis that immunotherapy does not add much to sorafenib as first-line therapy (Gollob *et al*, 2007; Ryan *et al*, 2007; Bracarda *et al*, 2008; Jonasch *et al*, 2010). In addition, the combination of sunitinib and IFN-*α* was studied in a phase I trial and showed no evidence of a therapeutic synergistic effect and displayed a poor safety profile (Motzer *et al*, 2009).

In conclusion, in the present study, the combination of sorafenib plus IL-2 did not improve the clinical efficacy of sorafenib monotherapy as first-line treatment of mRCC. Subgroup analysis suggested that patients with clear-cell histology and those with good prognosis receiving higher doses of IL-2 may respond best to the combination therapy.

## Figures and Tables

**Table 1:** Patient characteristics at baseline (intent-to-treat population)

	**Sorafenib+IL-2 (*n*=66)**	**Sorafenib (*n*=62)**
*Age at randomisation (years)*		
Median (interquartile range)	64 (57–69)	62 (52–69)
		
*Gender,* n (%)		
Male	52 (79)	43 (69)
Female	14 (21)	19 (31)
		
*Tumor stage at diagnosis,* n (%)		
I	5 (8)	3 (5)
II	17 (26)	10 (16)
III	14 (21)	24 (39)
IV	28 (42)	25 (40)
Missing	2 (3)	0
		
*MSKCC risk group,* n (%)		
Low	36 (55)	34 (55)
Intermediate	27 (41)	24 (39)
High	3 (5)	4 (6)
		
*Histological type,* n (%)		
Clear cell	58 (88)	56 (90)
Non-clear cell	8 (12)	6 (10)
		
*Previous nephrectomy,* n (%)		
No	18 (27)	16 (26)
Yes	48 (73)	46 (74)
		
*Sites of disease,* n (%)		
Lung	20 (30)	9 (15)
Liver	1 (2)	3 (5)
Lymph nodes	7 (11)	10 (16)
Kidney	1 (2)	1 (2)
Bone	2 (3)	3 (5)
Other site	4 (6)	4 (6)
Multiple sites	31 (47)	32 (52)

Abbreviations: IL-2=interleukin 2; MSKCC=Memorial Sloan Kettering Cancer Centre.

**Table 2:** Adverse events (⩾5% in either treatment group)

	**Sorafenib+IL-2 (*n*=66)**		**Sorafenib (*n*=62)**	
**Adverse event**	**Any grade**	**Grade ⩾3**	**Any grade**	**Grade ⩾3**
Fatigue, *n* (%)	12 (19)	2 (3)	10 (16)	1 (2)
Chest Pain, *n* (%)	3 (5%)	1 (2)	0	0
Influenza-like illness, *n* (%)	8 (12)	(2)	0	0
Pyrexia, *n* (%)	13 (20)	0	1 (2)	0
Anemia, *n* (%)	3 (5)	0	5 (8)	0
Neutropenia, *n* (%)	4 (6)	1 (2)	0	0
Hypertension, *n* (%)	6 (9)	1 (2)	10 (16)	4 (6)
Diarrhea, *n* (%)	15 (23)	0	17 (27)	0
Hemorrhoids, *n* (%)	1 (2)	0	4 (6)	0
Stomatitis, *n* (%)	16 (24)	3 (5)	7 (11)	1 (2)
Nausea, *n* (%)	3 (5)	0	3 (5)	1 (2)
Hand–foot skin reaction, *n* (%)	27 (41)	8 (12)	32 (52)	6 (10)
Alopecia, *n* (%)	4 (6)	0	4 (6)	0
Pruritus, *n* (%)	3 (5)	0	4 (6)	0
Piastrinopenia, *n* (%)	2 (3)	0	4 (6)	0
Anorexia, *n* (%)	3 (5)	0	1 (2)	0
Hypophosphatemia, *n* (%)	4 (6)	1 (2)	3 (5)	0
Blood amylase increase, *n* (%)	1 (2)	0	3 (5)	0
Blood creatinine increased, *n* (%)	1 (2)	0	3 (5)	0
Transaminase increase, *n* (%)	0	0	3 (5)	1 (2)
Hyperuricemia, *n* (%)	4 (6)	0	6 (10)	0
Arthralgia, *n* (%)	5 (8)	0	0	0
Dyspnoea, *n* (%)	5 (8)	0	1 (2)	1 (2)

Abbreviation: IL-2=interleukin 2.

## References

[bib1] Akaza H, Tsukamoto T, Onishi T, Miki T, Kinouchi T, Naito S (2006) A low-dose combination therapy of interleukin-2 and interferon-alpha is effective for lung metastasis of renal cell carcinoma: a multicenter open study. Int J Clin Oncol 11: 434–440 17180511 10.1007/s10147-006-0596-z

[bib2] Amagai Y, Matsumoto M, Hojo K, Iguchi M, Wada T, Tanaka H, Ide N, Kato A, Shichijo M, Abe K (2010) Combination therapy of interleukin-2 and sorafenib improves survival benefits and prevents spontaneous pulmonary metastasis in murine renal cell carcinoma models. Jpn J Clin Oncol 40: 503–507 20106881 10.1093/jjco/hyp200

[bib3] Bracarda S, Porta C, Boni C, Santoro A, Artioli F, Contu A, Mentuccia R, Gasparro D, Caserta C, De Angelis V, on behalf of GOIRC study group (2008) Randomized prospective phase 2 trial of two schedules of sorafenib daily and interferon-a2a (IFN) in metastatic renal cell carcinoma (mRCC) (RAPSODY) Pro ASCO GU 2008, abstract 357

[bib4] Escudier B, Eisen T, Stadler WM, Szczylik C, Oudard S, Siebels M, Negrier S, Chevreau C, Solska E, Desai AA, Rolland F, Demkow T, Hutson TE, Gore M, Freeman S, Schwartz B, Shan M, Simantov R, Bukowski RM, TARGET Study Group (2007a) Sorafenib in advanced clear-cell renal-cell carcinoma. N Engl J Med 356: 125–134 17215530 10.1056/NEJMoa060655

[bib5] Escudier B, Pluzanska A, Koralewski P, Ravaud A, Bracarda S, Szczylik C, Chevreau C, Filipek M, Melichar B, Bajetta E, Gorbunova V, Bay JO, Bodrogi I, Jagiello-Gruszfeld A, Moore N, AVOREN Trial investigators (2007b) Bevacizumab plus Interferon *α*-2a for treatment of metastatic renal cell carcinoma: a randomized double-blind phase III trial. Lancet 370: 2103–2111 18156031 10.1016/S0140-6736(07)61904-7

[bib6] Escudier B, Szczylik C, Hutson TE, Demkow T, Staehler M, Rolland F, Negrier S, Laferriere N, Scheuring UJ, Cella D, Shah S, Bukowski RM (2009) Randomized phase 2 trial of first line treatment with sorafenib *vs* interferon Alfa-2a in patients with metastatic renal cell carcinoma. J Clin Oncol 10: 1280–1289 10.1200/JCO.2008.19.3342 19171708

[bib7] Evans S, Day S, Royston P (2010) Minim program. Freely available at: http://www-users.york.ac.uk/~mb55/guide/minim.htm (accessed 14 Septemebr 2010)

[bib8] Fisher RI, Rosenberg SA, Fyfe G (2000) Long-term survival update for high-dose recombinant interleukin-2 in patients with renal cell carcinoma. Cancer J Sci Am 6(Suppl 1): S55–S57 10685660

[bib9] Gollob JA, Rathmell WK, Richmond TM, Marino CB, Miller EK, Grigson G, Watkins C, Gu L, Peterson BL, Wright JJ (2007) Phase 2 trial of sorafenib plus interferon alfa-2b as first- or second-line therapy in patients with metastatic renal cell cancer. J Clin Oncol 25: 3288–3295 17664476 10.1200/JCO.2007.10.8613

[bib10] Hudes G, Carducci M, Tomczak P, Dutcher J, Figlin R, Kapoor A, Staroslawska E, Sosman J, McDermott D, Bodrogi I, Kovacevic Z, Lesovoy V, Schmidt-Wolf IG, Barbarash O, Gokmen E, O’Toole T, Lustgarten S, Moore L, Motzer RJ, Global ARCC Trial (2007) Temsirolimus, interferon alfa, or both for advanced renal-cell carcinoma. N Engl J Med 356: 2271–2281 17538086 10.1056/NEJMoa066838

[bib11] Iguchi M, Matsumoto M, Hojo K, Wada T, Matsuo Y, Arimura A, Abe K (2009) Antitumor efficacy of recombinant human interleukin-2 combined with sorafenib against mouse renal cell carcinoma. Jpn J Clin Oncol 39: 303–309 19336449 10.1093/jjco/hyp021

[bib12] Jonasch E, Corn P, Pagliaro LC, Warneke CL, Johnson MM, Tamboli P, Ng C, Aparicio A, Ashe RG, Wright JJ, Tannir NM (2010) Upfront randomized phase 2 trial of sorafenib *vs* sorafenib and low-dose interferon alfa in patients with advanced renal cell carcinoma: clinical and biological analysis. Cancer 116: 57–65 19862815 10.1002/cncr.24685 PMC4636203

[bib13] McDermott DF, Atkins M (2008) Immunotherapy of metastatic renal cell carcinoma. Cancer J 14: 320–324 18836337 10.1097/PPO.0b013e31818675c4

[bib14] McDermott DF, Regan MM, Clark JI, Flaherty LE, Weiss GR, Logan TF, Kirkwood JM, Gordon MS, Sosman JA, Ernstoff MS, Tretter CP, Urba WJ, Smith JW, Margolin KA, Mier JW, Gollob JA, Dutcher JP, Atkins MB (2005) Randomized phase III trial of high-dose interleukin-2 *vs* subcutaneous interleukin-2 and interferon in patients with metastatic renal cell carcinoma. J Clin Oncol 23: 133–141 15625368 10.1200/JCO.2005.03.206

[bib15] Melichar B, Koralewski P, Ravaud A, Pluzanska A, Bracarda S, Szczylik C, Chevreau C, Filipek M, Delva R, Sevin E, Négrier S, McKendrick J, Santoro A, Pisa P, Escudier B (2008) First-line bevacizumab combined with reduced dose interferon-alpha2a is active in patients with metastatic renal cell carcinoma. Ann Oncol 19: 1470–1476 18408224 10.1093/annonc/mdn161

[bib16] Miyake H, Kurahashi T, Takenaka A, Inoue T, Fujisawa M (2009) Clinical outcome of combined immunotherapy with interferon-alpha and low-dose interleukine-2 for Japanese patients with metastatic renal cell carcinoma. Urol Oncol 27: 598–603 18818106 10.1016/j.urolonc.2008.07.023

[bib17] Motzer RJ, Escudier B, Oudard S, Hutson TE, Porta C, Bracarda S, Grünwald V, Thompson JA, Figlin RA, Hollaender N, Urbanowitz G, Berg WJ, Kay A, Lebwohl D, Ravaud A, RECORD-1 Study Group (2008) Efficacy of everolimus in advanced renal cell carcinoma: a double-blind, randomised, placebo-controlled phase III trial. Lancet 372: 427–429 18653228 10.1016/S0140-6736(08)61039-9

[bib18] Motzer RJ, Hudes G, Wilding G, Schwartz LH, Hariharan S, Kempin S, Fayyad R, Figlin RA (2009) Phase 1 trial of sunitinib malate plus interferon-alpha for patients with metastatic renal cell carcinoma. Clin Genitourin Cancer 7: 28–33 19213665 10.3816/CGC.2009.n.005 PMC3394091

[bib19] Motzer RJ, Hutson TE, Tomczak P, Michaelson MD, Bukowski RM, Rixe O, Oudard S, Negrier S, Szczylik C, Kim ST, Chen I, Bycott PW, Baum CM, Figlin RA (2007) Sunitinib *vs* interferon-alfa in metastatic renal-cell carcinoma. N Engl J Med 356: 115–124 17215529 10.1056/NEJMoa065044

[bib20] Negrier S, Escudier B, Lasset C, Douillard JY, Savary J, Chevreau C, Ravaud A, Mercatello A, Peny J, Mousseau M, Philip T, Tursz T (1998) Recombinant human interleukin-2, recombinant human interferon alfa-2a, or both in metastatic renal cell carcinoma. N Engl J Med 338: 1272–1278 9562581 10.1056/NEJM199804303381805

[bib21] Negrier S, Perol D, Ravaud A, Chevreau C, Bay JO, Delva R, Sevin E, Caty A, Escudier B, For The French Immunotherapy Intergroup (2007) Medroxyprogesterone, interferon alfa 2a, interleukin 2, or combination of both cytokines in patients with metastatic renal cell carcinoma of intermediate prognosis: results of a randomized controlled trial. Cancer 110: 2468–2477 17932908 10.1002/cncr.23056

[bib22] Rini BI, Halabi S, Rosenberg JE, Stadler WM, Vaena DA, Archer L, Atkins JN, Picus J, Czaykowski P, Dutcher J, Small EJ (2010) Phase 3 trial of bevacizumab plus interferon alfa *vs* interferon alfa monotherapy in patients with metastatic renal cell carcinoma: final results of CALGB 90206. J Clin Oncol 28: 2137–2143 20368558 10.1200/JCO.2009.26.5561 PMC2860433

[bib23] Rosenberg SA, Yang JC, Topalian SL, Schwartzentruber DJ, Weber JS, Parkinson DR, Seipp CA, Einhorn JH, White DE (1994) Treatment of 283 consecutive patients with metastatic melanoma or renal cell cancer using high-dose bolus interleukin 2. JAMA 271: 908–1013 8120958

[bib24] Ryan CW, Goldman BH, Lara Jr PN, Mack PC, Beer TM, Tangen CM, Lemmon D, Pan CX, Drabkin HA, Crawford ED, Soutwest Oncology Group (2007) Sorafenib with interferon alfa-2b as first line treatment of advanced renal carcinoma: a phase 2 study of the Southwest Oncology Group. J Clin Oncol 25: 3296–3301 17664477 10.1200/JCO.2007.11.1047

[bib25] Simon RM, Steinberg SM, Hamilton M, Hildesheim A, Khleif S, Kwak LW, Mackall CL, Schlom J, Topalian SL, Berzofsky JA (2001) Clinical trial designs for the early clinical development of therapeutic cancer vaccines. J Clin Oncol 19: 1848–1854 11251017 10.1200/JCO.2001.19.6.1848

[bib26] Sternberg CN, Davis ID, Mardiak J, Szczylik C, Lee E, Wagstaff J, Barrios CH, Salman P, Gladkov OA, Kavina A, Zarbá JJ, Chen M, McCann L, Pandite L, Roychowdhury DF, Hawkins RE (2010) Pazopanib in locally advanced or metastatic renal cell carcinoma: results of a randomized phase 3 trial. J Clin Oncol 28: 1061–1068 20100962 10.1200/JCO.2009.23.9764

[bib27] Therasse P, Arbuck SG, Eisenhauer EA, Wanders J, Kaplan RS, Rubinstein L, Verweij J, Van Glabbeke M, van Oosterom AT, Christian MC, Gwyther SG (2000) New guidelines to evaluate the response in solid tumors: European Organization for Research and Treatment of Cancer, National Cancer Institute of the United States, National Cancer Institute of Canada. J Natl Cancer Inst 92: 205–216 10655437 10.1093/jnci/92.3.205

[bib28] Wilhelm SM, Carter C, Tang L, Wilkie D, McNabola A, Rong H, Chen C, Zhang X, Vincent P, McHugh M, Cao Y, Shujath J, Gawlak S, Eveleigh D, Rowley B, Liu L, Adnane L, Lynch M, Auclair D, Taylor I, Gedrich R, Voznesensky A, Riedl B, Post LE, Bollag G, Trail PA (2004) BAY 43-9006 exhibits broad spectrum oral anti-tumor activity and targets the Raf/MEK/ERK pathway and receptor tyrosine kinases involved in tumor progression and angiogenesis. Cancer Res 64: 7099–7109 15466206 10.1158/0008-5472.CAN-04-1443

[bib29] Yang JC, Sherry RM, Steinberg SM, Topalian SL, Schwartzentruber DJ, Hwu P, Seipp CA, Rogers-Freezer L, Morton KE, White DE, Liewehr DJ, Merino MJ, Rosenberg SA (2003) Randomized study of high-dose and low-dose interleukin-2 in patients with metastatic renal cancer. J Clin Oncol 21: 3127–3132 12915604 10.1200/JCO.2003.02.122 PMC2275327

